# Antisense oligonucleotide targeting CD39 improves anti-tumor T cell immunity

**DOI:** 10.1186/s40425-019-0545-9

**Published:** 2019-03-12

**Authors:** Abhishek S. Kashyap, Tamara Thelemann, Richard Klar, Sandra M. Kallert, Julia Festag, Melanie Buchi, Lisa Hinterwimmer, Monika Schell, Sven Michel, Frank Jaschinski, Alfred Zippelius

**Affiliations:** 1 Cancer Immunology, Department of Biomedicine, University Hospital Basel, Basel, Switzerland; 2 Secarna Pharmaceuticals GmbH, Planegg/Martinsried, Germany; 3 Medical Oncology, University Hospital Basel, Basel, Switzerland; 4 Present address: Novartis Institute of Biomedical Research, 4002 Basel, Switzerland

**Keywords:** CD39, CD73, Ectonucleotidase, Antisense oligonucleotide (ASO), Immunotherapy, ATP, Adenosine

## Abstract

**Background:**

Cancer cells are known to develop mechanisms to circumvent effective anti-tumor immunity. The two ectonucleotidases CD39 and CD73 are promising drug targets, as they act in concert to convert extracellular immune-stimulating ATP to adenosine. CD39 is expressed by different immune cell populations as well as cancer cells of different tumor types and supports the tumor in escaping immune recognition and destruction. Thus, increasing extracellular ATP and simultaneously reducing adenosine concentrations in the tumor can lead to effective anti-tumor immunity.

**Methods:**

We designed locked nucleic acid (LNA)-modified antisense oligonucleotides (ASOs) with specificity for human or mouse CD39 that do not need a transfection reagent or delivery system for efficient target knockdown. Knockdown efficacy of ASOs on mRNA and protein level was investigated in cancer cell lines and in primary human T cells. The effect of CD39 knockdown on ATP-degrading activity was evaluated by measuring levels of ATP in tumor cell supernatants and analysis of T cell proliferation in the presence of extracellular ATP. The in vivo effects of CD39-specific ASOs on target expression, anti-tumor immune responses and on tumor growth were analyzed in syngeneic mouse tumor models using multi-color flow cytometry.

**Results:**

CD39-specific ASOs suppressed expression of CD39 mRNA and protein in different murine and human cancer cell lines and in primary human T cells. Degradation of extracellular ATP was strongly reduced by CD39-specific ASOs. Strikingly, CD39 knockdown by ASOs was associated with improved CD8^+^ T cell proliferation. Treatment of tumor-bearing mice with CD39-specific ASOs led to dose-dependent reduction of CD39-protein expression in regulatory T cells (Tregs) and tumor-associated macrophages. Moreover, frequency of intratumoral Tregs was substantially reduced in CD39 ASO-treated mice. As a consequence, the ratio of CD8^+^ T cells to Tregs in tumors was improved, while PD-1 expression was induced in CD39 ASO-treated intratumoral CD8^+^ T cells. Consequently, CD39 ASO treatment demonstrated potent reduction in tumor growth in combination with anti-PD-1 treatment.

**Conclusion:**

Targeting of CD39 by ASOs represents a promising state-of-the art therapeutic approach to improve immune responses against tumors.

**Electronic supplementary material:**

The online version of this article (10.1186/s40425-019-0545-9) contains supplementary material, which is available to authorized users.

## Background

Local immunosuppression in the tumor microenvironment is a hallmark of many cancers. Reinvigoration of T cell function by checkpoint blockade can result in striking clinical responses, but is effective only in a minority of patients. Immunosuppressive pathways operational in tumors significantly impact the efficacy of immunotherapy. The adenosine pathway is one of these immunosuppressive pathways. CD39 and CD73 are ectonucleotidases which act in concert to degrade ATP to immunosuppressive adenosine [1–4]. Binding of adenosine to A2A or A2B receptors on T cells and natural killer (NK) cells results in dampened proliferation and cytolytic functions leading to immunosuppression. Adenosine induces alternate polarization of tumor associated macrophages (TAMs) towards immunosuppressive M2-like TAMs capable of secreting increased amounts of IL-10 and reduced pro-inflammatory cytokines [4, 5]. Mice lacking CD39 show improved tumor rejection [1], making this pathway a promising therapeutic target [6]. Dying cancer cells release ATP which may enhance anti-tumor immune responses e.g. through recruitment and activation of dendritic cells (DCs), macrophages and their precursors [7, 8]. Binding of ATP to P_2_X_7_ receptors on DCs results in DC activation and the release of pro-inflammatory cytokines such as IL-1β or IL-18 [9]. These cytokines in turn activate NK cells, T cells, and macrophages and enhance their proliferation and cytolytic functions [5]. Co-expression of CD73 by tumor cells or tumor-associated stromal cells leads to further degradation to adenosine that accumulates in the tumor microenvironment [4]. Taken together, targeting CD39 will decrease the degradation of ATP and therefore result in increased levels of immune stimulatory extracellular ATP. This will concurrently lead to the suppression of adenosine through the prevention of AMP generation, the substrate of CD73.

CD39 is widely expressed by different immune cells such as monocytes, neutrophils, macrophages, B lymphocytes, DCs, as well as some subsets of NK cells, and T cells [3]. Tregs express CD39 and CD73, which enable them to generate adenosine leading to immunosuppression. In addition, enhanced CD39 expression has been described in various cancer cells of kidney, lung, testicular and thyroid tumors as well as in lymphoma [7].

In the current study we targeted CD39 expression with locked nucleic acid (LNA) Gapmer antisense oligonucleotides (ASOs). LNA Gapmers are ASOs with a length of typically 14–17 nucleotides. They contain a central “gap” of DNA monomers flanked by LNA-modified nucleotides. LNA modifications result in increased target affinity. The central DNA “gap” recruits RNase H, which cleaves the target RNA upon binding. LNA Gapmers have fully phosphorothioated (PTO) backbones, which ensure resistance to enzymatic degradation [10]. In contrast to earlier chemical modifications, LNA Gapmers do not require transfection reagents or conjugations for efficient gene silencing in vitro [11, 12], a process called gymnosis. In vivo unformulated and unconjugated LNA Gapmers achieve specific target knockdown in several tissues including tumors after systemic administration [12].

Here we demonstrate that unformulated human (h)CD39 specific ASOs achieved potent target knockdown in vitro, reduced degradation of extracellular ATP by T cells, and reverted growth suppression in T cells caused by treatment with ATP. In syngeneic mouse tumor models, systemic treatment with murine (m)CD39-specific ASO resulted in potent knockdown of CD39 expression in specific immune cell populations, namely Tregs and TAMs, as well as in a reduction of the frequency of intratumoral Tregs. Moreover, tumor growth was significantly reduced when CD39-specific ASO was combined with an anti-PD-1 antibody.

## Methods

### Antisense oligonucleotides

15-, 16- and 17-mer ASOs were selected based on the human NM_001776 or murine NM_001304721 CD39 mRNA (encoded by the ENTPD1 gene). Main criterion for sequence selection was selectivity to avoid undesired off-target effects. LNA modified Gapmers were ordered from Exiqon or Eurogentec and dissolved in H_2_O (stock concentration: 1 mM). Antisense oligonucleotides were added to cells without the use of a transfection reagent in vitro and without any delivery system in vivo.

Sequences of ASOs and control oligonucleotides used in the study:

**Table:** 

**ASO ID**	**Length**	**Sequence**	**Description**
A04019H	15	+G* + T* + A*A*G*C*C*C*T*G*A*T* + G* + T* + T	Human specific ASO
A04040H	16	+G* + T* + T*T*G*T*G*T*G*A*G*A*G*C* + T* + T	Human specific ASO, used for in vitro experiments
A04042H	17	+T* + G* + C*C*A*G*A*G*T*G*C*C*T*G* + A* + T* + C	Human specific ASO
A04044H	17	+T* + T* + A*C*G*T*T*C*A*C*T*A*C*C* + T* + T* + C	Human specific ASO
A04045H	17	+C* + A* + C*T*T*A*C*G*T*T*C*A*C*T* + A* + C* + C	Human specific ASO
A04011MR	16	+A* + G* + T*A*A*T*C*C*A*C*C*C*A* + T* + A* + G	Mouse specific ASO, used for in vivo experiments, named: “CD39 ASO” in the text
Control oligo 1	18	+C* + G* + T*T*T*A*G*G*C*T*A*T*G*T*A* + C* + T* + T	Control oligonucleotide, reference: PMID: 26072406, used for in vitro and in vivo experiments
Control oligo 2	17	+T* + C* + T*A*T*C*G*T*G*A*T*G*T*T* + T* + C* + T	Control oligonucleotide, used for in vitro experiments

+ indicates LNA-modified nucleotides and * indicates PTO linkages

### Cell culture medium and supplements

RPMI supplemented with antimycotic-antibiotic (100x) and sodium pyruvate (100x) were used at 1% and heat inactivated (30 min at 56 °C) FBS was used at 10% for cell culture experiments (RPMIfs). Cell culture reagents were obtained from GIBCO.

### Blood samples

PBMC were derived from Buffy Coats purchased from *Blutspendedienst des Bayerischen Roten Kreuzes gemeinnützige GmbH; Hauptverwaltung München; Vertrieb; Herzog-Heinrich-Straße 2; 80336 München* or obtained from leukapheresis products.

### Mice

C57BL/6 and Balb/c mice were bred in-house at University Hospital Basel, Switzerland. In case of unavailability, mice were also obtained from Janvier Labs (France). Animals were housed under specific pathogen-free conditions. All animal experiments were performed in accordance with Swiss federal regulations. Sex-matched littermates at 8–12 weeks of age at start of experiments were used.

### Quantigene mRNA expression analysis

Target expression on mRNA level was determined using bDNA assay (QuantiGene SinglePlex Assay Kit 96-Well plate format and QuantiGene Sample Processing Kit for cultured cells, Thermo Fisher Scientific). The following probe sets were used: human ENTPD1 (SA-11803); human HPRT1 (SA-10030); mouse ENTPD1, (SB-13732); mouse HPRT1 (SB-15463). All reagents were purchased from Affymetrix/Thermo Fisher Scientific.

### FACS staining for surface proteins for human samples

Cells were spun down at 500 g for 5 min, and washed in FACS buffer (1x PBS, 5% FBS) followed by incubation for 25 min at 4 °C in 50 μl FACS buffer per well in 96-well U-bottom plates containing the respective antibodies (anti- human CD8 (clone RPA-T8), anti-human CD4 (clone RPA-T4), anti-human CD39 (clone A1), mouse IgG,κ isotype control and 7-AAD (all from BioLegend). Subsequently, cells were washed twice with FACS buffer and analyzed on a NovoCyte Flow Cytometer (ACEA Biosciences, Inc.).

### hCD39 protein expression in human CD8^+^ or CD4^+^ T cells upon oligonucleotide treatment

CD4^+^ and CD8^+^ T cells were separately isolated from PBMCs using MACS (Miltenyi, according to the manufacturer’s instructions). CD4^+^ or CD8^+^ T cells (100,000 per well) were plated on anti-CD3-coated (2 μg/ml; clone OKT3; eBioscience) 96-well U-bottom plates in RPMIfs supplemented with anti-CD28 (2 μg/ml; clone CD28.2; eBioscience) and IL-2 (60 IU/ml; Peprotec) and treated with 5 μM of oligonucleotides for a total treatment time of six days without the use of a transfection reagent. Activation medium and oligonucleotides were replaced after three days. As mock control, cells were cultivated in activation medium without oligonucleotide. On day six after start of treatment, cells were transferred to uncoated 96-well U-bottom plates and cultivated in cell culture medium supplemented with IL-2 (20 IU/ml) in the absence of oligonucleotides. Cells were split 1:2 every third day. hCD39 protein expression was analyzed on day three, six and eleven after removal of oligonucleotides by flow cytometry.

### Labelling of cells with proliferation dye

T cells were isolated as described above. Cells were washed twice with PBS, resuspended and adjusted to 2x of the desired final concentration in PBS (pre-warmed to room temperature). A 20 μM solution of cell proliferation dye eFluor™ 450 (eBioscience) in PBS was prepared (pre-warmed to room temperature) and mixed 1:1 with the 2x cell suspension while gently vortexing. Cells were incubated for 10 min at 37 °C in the dark. The labelling reaction was stopped by adding 4–5 volumes of cold complete medium (containing 10% FBS) and cells were incubated on ice for 5 min. Cells were washed 3 times with complete medium before further culturing.

### Quantification of extracellular ATP levels in cell supernatants of oligonucleotide-treated human CD8^+^ T cells

CD8^+^ T cells were isolated from PBMC as described above. 100,000 cells per well were plated on anti-CD3-coated 96 well U-bottom plates in activation medium (as described above) and treated with 5 μM of oligonucleotides for a total treatment time of six days without the use of a transfection reagent. Activation medium and oligonucleotides were replaced after three days. As mock control, cells were cultivated in activation medium without oligonucleotide. Six days after treatment start, cell culture medium was supplemented with ATP (SIGMA-Aldrich) at a concentration of 2 µM. After an incubation time of 30 min, residual ATP concentration in cell culture supernatants or cell-free medium was determined using the ATP Bioluminescence Assay Kit HSII (Roche) according to the manufacturer’s protocol.

### Assessment of extracellular ATP on proliferation of oligonucleotide-treated human CD8^+^ T cells

CD8^+^ T cells were isolated and labelled with cell proliferation dye as described above. Cells (100,000 per well) were plated on anti-CD3-coated 96 well U-bottom plates in activation medium and treated with oligonucleotides at a concentration of 5 μM for a total treatment time of five days without the use of a transfection reagent. Activation medium and oligonucleotides were replaced after three days. On day three and day four after start of oligonucleotide treatment, 400 μM of ATP or vehicle were added to cells. The following day, hCD39 protein expression, proliferation, and absolute numbers of CD8^+^ T cells were analyzed by flow cytometry (123count eBeads from eBioscience were used to obtain absolute counts). Proliferation index was calculated using the formula: $$ \raisebox{1ex}{${\sum}_0^i{N}_i$}\!\left/ \!\raisebox{-1ex}{${\sum}_0^i{N}_i/2$}\right. $$, whereas i is the generation number and N is the absolute cell count in the respective generation.

### In vivo tumor challenge and treatment protocol

C57BL/6 mice were injected subcutaneously into the right flank with 500,000 syngeneic murine MC38 colorectal adenocarcinoma cells (kindly provided by Thomas Wirth, Medizinischen Hochschule Hannover) suspended in phenol red-free DMEM (without additives). Once tumors reached an average volume of 60–80 mm^3^ (day 12–15), mice were injected intraperitoneally with 200 μl of PBS suspended solutions of CD39 ASO at indicated doses, non-targeting control oligonucleotide (control oligo 1) (100 mg/kg) or left untreated. On day 9 post first compound injection, mice were euthanized and tumors, and in selected cases tumor draining lymph nodes were excised and processed for FACS analyses as outlined below. For tumor growth experiments EMT6 (obtained from ATCC) murine breast cancer cells were injected into the mammary gland of female Balb/c mice. Once tumors reached an average volume of 80 mm^3^ (Day 8), mice were injected intraperitoneally with 200 μl of PBS suspended solutions of CD39 ASO (20 mg/kg), non-targeting control oligonucleotide (control oligo 1) (20 mg/kg) and/or mouse anti-PD-1 (12.5 mg/kg) (RPM1–14, rat IgG2a, BioXCell) at indicated timepoints. Tumor volume was calculated according to the formula: D /2*d*d, with D and d being the longest and shortest tumor diameter in mm, respectively.

### Phenotypic characterization of tumor infiltrating and lymph node cells by flow cytometry

Tumors were harvested from tumor bearing mice and minced using razor blades followed by digestion with accutase (PAA), collagenase IV (Worthington), hyaluronidase (Sigma), and DNAse type IV (Sigma) for 60 min at 37 °C with constant shaking. The cell suspensions were filtered using a cell strainer (70 μm). Lymph node cells were isolated by mashing using the end of a 1 mL syringe. Cells were filtered through a 70 μm nylon mesh. Red blood cells (RBCs) were lysed using RBC lysis buffer (eBioscience). Single cell suspensions derived from tumor and lymph nodes were blocked with rat anti-mouse FcγIII/II receptor (CD16/CD32) blocking antibodies (“Fc-Block”) and stained with live/dead cell-exclusion dye (Zombie UV dye; Biolegend). The cells were then incubated with fluorophore-conjugated antibodies directed against cell surface antigens, washed and resuspended in FACS buffer (PBS + 2%FBS). For intracellular antigens (FoxP3), cells stained with cell surface antibodies were fixed (IC fix, eBioesceince) and permeabilized (Perm buffer; eBioscience) prior to incubation with antibodies directed against intracellular antigens. Cell populations were analyzed on a BD Fortessa. Cells were discriminated using the following combination of cell markers after gating on single cells (discriminated by FSC-A and FSC-H) and excluding non-viable cells (Live/Dead negative). TAMs were denoted by CD45^+^CD11b^+^Ly6C^−^Ly6G^−^F4/80^high^. G-MDSC were CD45^+^CD11b^+^Ly6G^+^ and M-MDSC were CD45^+^CD11b^+^Ly6C^+^. CD4^+^/CD8^+^ T cells were CD45^+^CD11b^−^CD3^+^CD4^+^ or CD8^+^. Tregs were FOXP3^+^CD25^+^CD4^+^ T cells. B cells were CD45^+^CD11b^−^CD3^−^CD19^+^. Tumor cells were noted as CD45^−^.

### Statistical analysis

Statistical analysis was performed by GraphPad Prism 7.0 (GraphPad Software). If applicable, results are represented as mean +/− SD. Pairwise comparisons were analyzed using two-tailed student’s t-test and grouped analyses were performed using one-way non-parametric ANOVA with multiple comparison with Tukey’s post hoc test. *p*-values ≤0.05 (*); *p* ≤ 0.01 (**); *p* ≤ 0.001(***); *p* ≤ 0.0001 (****) were considered significant.

## Results

### CD39 protein expression is reduced in human CD4^+^ and CD8^+^ T cells after hCD39-specific ASO treatment

**Fig. 1:**
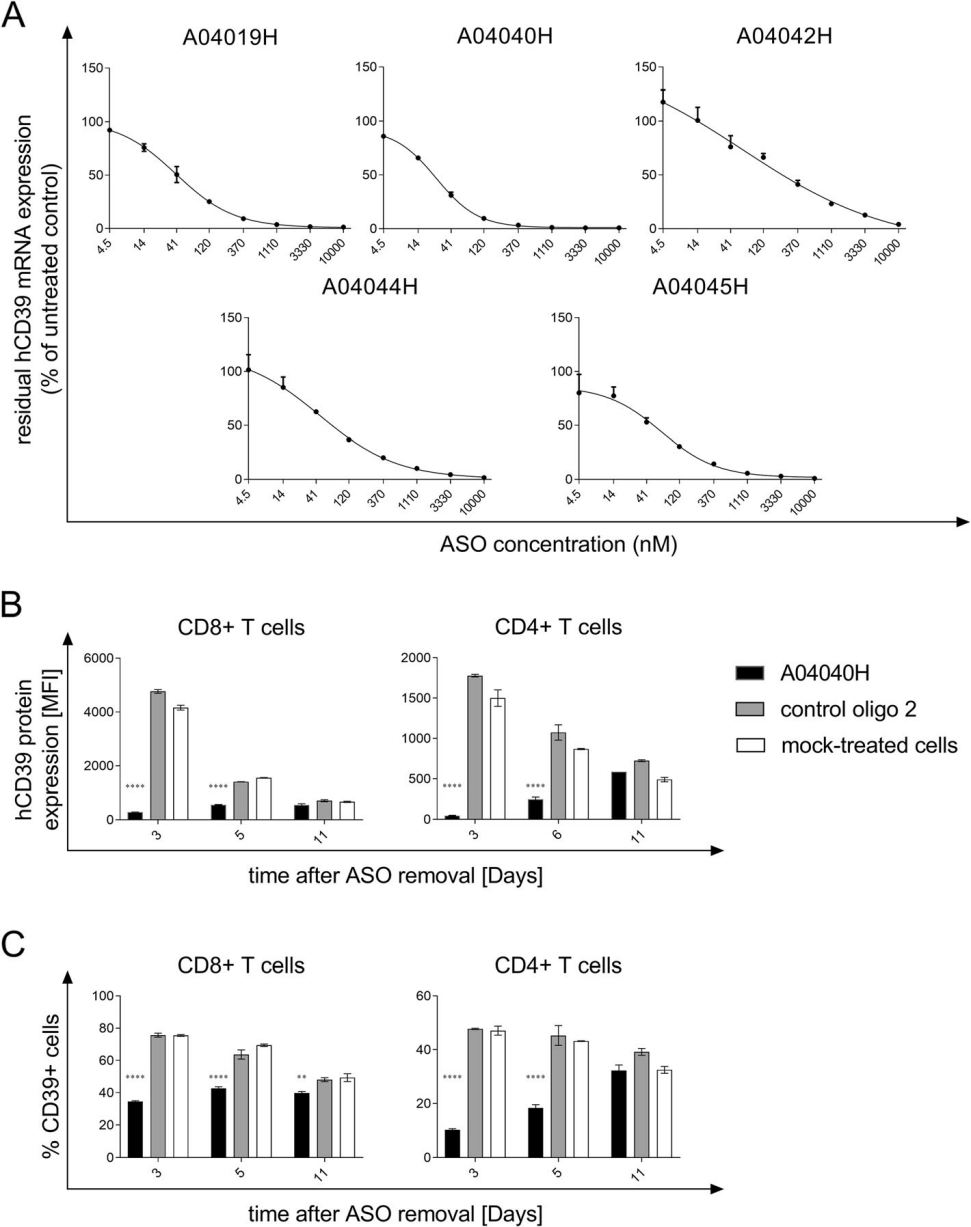
Selection of hCD39-specific ASO and assessment of target knockdown efficiency. (**a**) HDLM-2 cells were treated for three days with the indicated concentrations of the respective antisense oligonucleotide without the use of a transfection reagent. hCD39 mRNA expression values were normalized to expression of the housekeeping gene HPRT1. Residual hCD39 mRNA expression relative to untreated cells (set to 100%) is depicted. Depicted is the mean of triplicate wells +/− SD. (**b**) and (**c**) Human anti-CD3, anti-CD28, and IL-2 activated CD8^+^ and CD4^+^ T cells were treated with 5 μM of the hCD39-specific ASO A04040H or the control oligo 2 for a total treatment time of six days without the use of a transfection reagent. Thereafter, oligonucleotides were removed and cells were re-plated on plates not coated with anti-CD3. hCD39 protein expression was analyzed by flow cytometry three, six and eleven days after oligonucleotide removal. hCD39 protein expression is depicted as mean fluorescence intensity (MFI) and was calculated by subtracting the MFI of hCD39 by the MFI of unspecific isotype control (**b**) or as % CD39^+^ cells of alive cells (**c**). Data is shown as mean of duplicates +/−SD. Asterisks indicate significant differences compared to control oligo treatment within each time point

ASOs with specificity for hCD39 were initially screened for target mRNA suppression in HDLM-2 cells without the use of a transfection reagent, a human Hodgkin lymphoma cell line with high endogenous expression of CD39. The most potent candidates were further tested in dose-response experiments. Figure 1a depicts the concentration dependency of target knockdown for the ASOs with the highest activity. The corresponding IC50 values are shown in Additional file 1: Table S1. The most potent hCD39-specific ASO (A04040H, IC50 25.28 nM) was selected for subsequent experiments.

The potency of A04040H was investigated in human T cells. To this end, human T cells were exposed to A04040H for six days without the use of a transfection reagent and, upon washing, subsequently cultured in the absence of A04040H. CD39 protein expression was determined three, six and eleven days after oligonucleotide removal. CD39 protein expression in T cells remained downregulated up to 6 days after removal of A04040H as measured by median fluorescence intensity (Fig. 1b) and %CD39^+^ cells (Fig. 1c), respectively. Treatment with the control oligo 2 that has no sequence complementarity to any human or murine mRNA had no inhibitory effect on hCD39 protein expression as compared to mock-treated cells (Fig. 1b and c).

### hCD39-specific ASO prevents extracellular ATP degradation by human CD8^+^ T cells

**Fig. 2:**
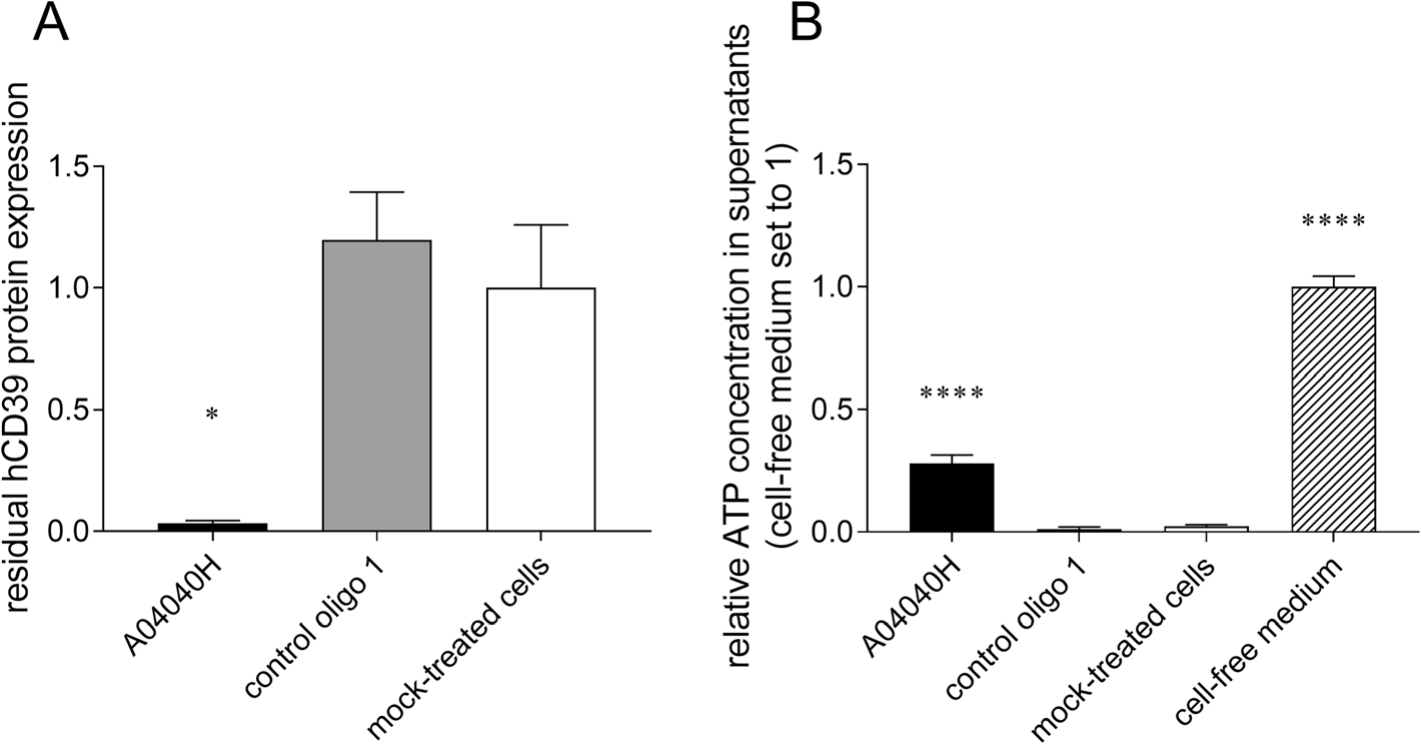
Decrease in CD39 expression and extracellular ATP accumulation in human CD8^+^ T cells in presence of hCD39-specific ASO. Human anti-CD3, anti-CD28, and IL-2 activated CD8^+^ T cells were treated with 5 μM of the hCD39 specific ASO A04040H or the control oligo 1 for a total treatment time of six days. hCD39 protein expression was analyzed by flow cytometry. (**a**) Residual hCD39 expression of oligonucleotide-treated cells relative to mock-treated cells (set to 1). (**b**) After six days of ASO treatment, 2 μM of ATP were added to cells or cell-free medium. ATP concentration in cell supernatants was determined after 30 min of incubation with ATP and is presented relative to cell-free medium (set to 1). Data is shown as mean of triplicates +/− SD. Asterisks indicate significant differences compared to control oligo treatment

We assessed the effects of hCD39-specific ASO on the capacity of CD8^+^ T cells and tumor cells (here Burkitt’s lymphoma cells) to degrade extracellular ATP, the substrate of CD39. Cells were treated with A04040H for six days without the use of a transfection reagent. ATP was added to cell culture supernatants or cell-free medium for 30 min after which ATP levels (and CD39 protein expression) were determined. As observed above, treatment with A04040H led to strong suppression of CD39 protein expression compared to control oligo 1 or mock-treated cells (Fig. 2a and Additional file 2: Figure S1A). In cells treated with A04040H, the decreased expression of CD39 correlated with significantly increased levels of extracellular ATP compared to control oligo 1 or mock treatment (Fig. 2b and Additional file 2: Figure S1B). This suggests that the hCD39-specific ASO prevents CD39-mediated degradation of extracellular ATP by targeting CD39 expression.

### hCD39-specific ASO reverts the impairment of T cell proliferation and viability caused by ATP by-products

**Fig. 3:**
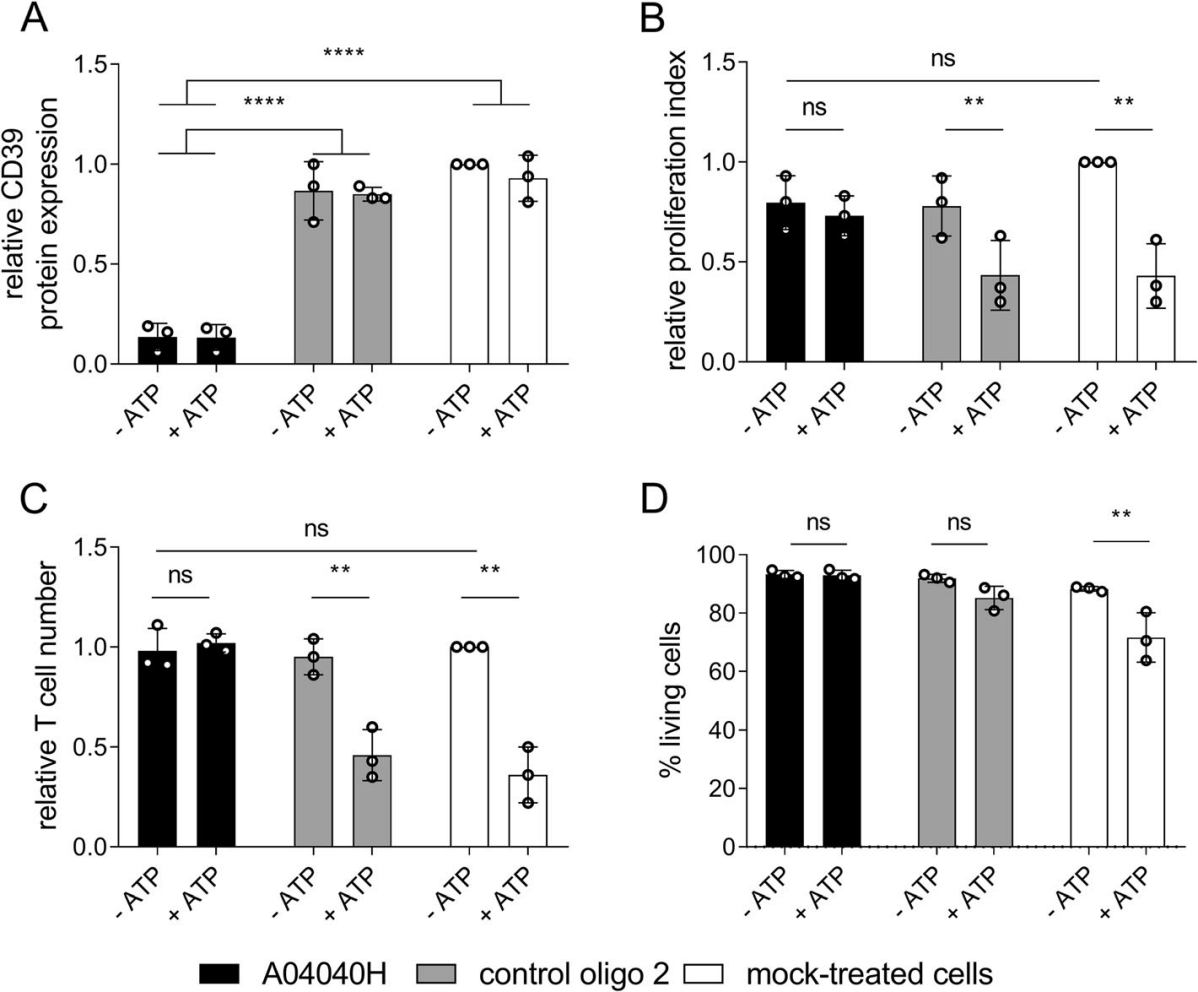
Improved human CD8^+^ T cell proliferation in the presence of hCD39-specific ASO and extracellular ATP. Human CD8^+^ T cells were labelled with cell proliferation dye, activated with anti-CD3, anti-CD28 and IL-2, and treated with 5 μM of the antisense oligonucleotide A04040H or the control oligo 2 for a total treatment time of five days. Subsequently, 400 μM of ATP was added to cells on day three and day four after start of oligonucleotide-treatment. On day five after start of oligonucleotide treatment, (**a**) hCD39 protein expression, (**b**) proliferation index, (**c**) relative cell numbers of CD8^+^ T cells relative to mock-treated cells without ATP (set as 1) as wells as (**d**) % living cells were analyzed using flow cytometry. Cells were derived from two different donors, processed on three different dates. Bar graphs depict the mean of three independent experiments run in triplicates +/− SD. Asterisks indicate significant differences between the respective conditions

We next investigated the effects of A04040H on CD8^+^ T cell proliferation in the presence or absence of extracellular ATP. A04040H treatment of CD8^+^ T cells potently suppressed CD39 protein expression (Fig. 3a). In the absence of extracellular ATP no significant differences in proliferation index (Fig. 3b) or absolute cell numbers (Fig. 3c) were observed compared to control oligo 2 or mock treatment. As expected, ATP supplementation reduced the proliferation (Fig. 3b) and reduced absolute numbers (Fig. 3c) of control oligo 2 or mock-treated CD8^+^ T cells. This was entirely rescued by treatment with A04040H. We furthermore observed no impact on cell viability upon exposure of A04040H-treated T cells with ATP (Fig. 3d). In contrast, there was a reduction in cell viability (non-significant) when ATP was added to control oligo 2-treated cells and a significant reduction in cell viability when ATP was added to mock-treated cells. In summary, these results reveal that A04040H treatment rescues CD39-induced suppression of T cell proliferation and cell viability, most likely by inhibiting ATP degradation.

### mCD39-specific ASO preferentially downregulates CD39 expression in murine tumor infiltrating Tregs and TAMs

**Fig. 4:**
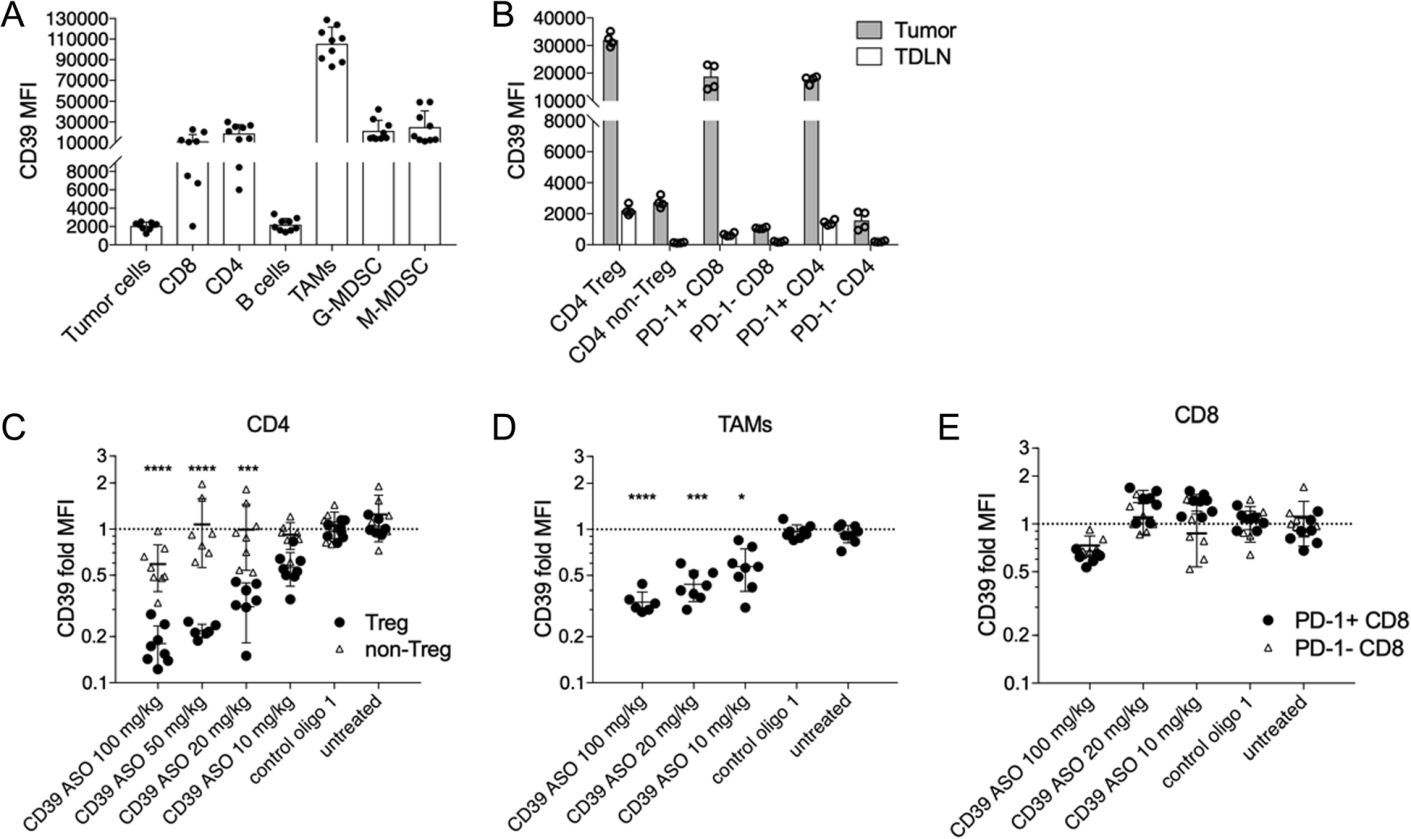
CD39 expression on various murine tumor infiltrating lymphocyte and myeloid populations. (**a**) CD39 expression on live CD45+ immune cells or CD45- tumor cells from freshly digested MC38 murine tumors (around 100 to 200 mm^3^) of untreated mice was assessed by flow cytometry. TAMs: CD11b^+^Ly6C^−^Ly6G^−^F4/80^+^; G-MDSC: CD11b^+^Ly6G^+^; M-MDSC: CD11b^+^Ly6C^+^. (**b**) CD39 expression on Tregs (CD4^+^CD25^+^FoxP3^+^) and non-Tregs (CD4^+^CD25^−^FoxP3^−^) as well as PD-1^+^ or PD-1^−^ CD4 and CD8 T cells was assessed in tumors and tumor draining-lymph nodes (TDLN) of untreated mice. (**c)**-(**e**) Mice bearing palpable tumors (50–80 mm^3^) were injected i.p with the indicated doses of CD39 ASO or with 100 mg/kg of control oligo 1. Day 9 post ASO injection tumors were digested and CD39 expression on tumor infiltrating Tregs/non-Tregs (**c**), TAMs (**d**) and CD8 (**e**) was assessed by flow cytometry. Data is represented as fold-change in CD39 MFI compared to control oligo. In all cases each data point represents a mouse. Pooled data from two to three independent repeats. Error bars indicate SD. Asterisks indicate significant differences compared to control oligo 1 group

We next utilized syngeneic tumor models to assess the in vivo effects of CD39 ASOs. Upon subcutaneous injection of MC38 colon adenocarcinoma cells, CD39 protein expression was initially assessed on tumor cells and tumor infiltrating immune populations. Myeloid cells, namely TAMs, as well as T lymphocytes expressed higher levels of CD39 compared to B cells and tumor cells (Fig. 4a). CD4^+^ Tregs as well as PD-1 expressing CD8^+^ and CD4^+^ T cell subsets had higher CD39 expression compared to non-Tregs and PD-1 negative cells, respectively (Fig. 4b). Of note, CD39 expression was higher in intratumoral T cells compared to T cells derived from the tumor draining lymph nodes (TDLN) (Fig. 4b).

To assess target downregulation and efficacy in vivo*,* murine-specific CD39 ASOs (Additional file 3: Table S2) were tested in vitro in mouse cancer cell line A20 (B cell lymphoma) from which the most potent mCD39-specific ASO A04011MR was selected as candidate (Additional file 4: Figure S2) for in vivo experiments. Systemic administration (intraperitoneal, i.p.) of the mCD39-specific ASO in MC38 tumor bearing mice resulted in a specific and dose-dependent downregulation of CD39 protein in tumor infiltrating CD4^+^ Tregs as well as TAMs (Fig. 4c, d and Additional file 5: Figure S3A). Greater than 50% of target downregulation was observed in Tregs, and TAMs, even at the low dose of 20 mg/kg of the ASO. CD39 expression in non-Tregs, CD8^+^ T cells, either PD-1^+^ or PD-1^−^, as well as tumor cells remained unchanged (Fig. 4c and e and Additional file 5: Figure S3B). When assessed for percentage of CD39 positive T cell populations, we observed a significant decrease in the number of CD39 expressing CD3^+^ T cells as well as CD4^+^ and Tregs in tumors of mice treated with CD39 ASO compared to control oligo 1 (Additional file 5: Figure S3C, D, F and G). Similar to that observed for CD39 surface expression (assessed by MFI), number of CD39^+^ CD8^+^ T cells in CD39 ASO mice remained non-significantly different to control oligo 1 (Additional file 5: Figure S3E). Together, these data suggest that systemically administered mCD39-specific ASO enters distant tumors and induces cell type specific downregulation of the target and the target cell populations.

### mCD39-specific ASO targets intratumoral Tregs and improves CD8^+^:Treg ratio

**Fig. 5:**
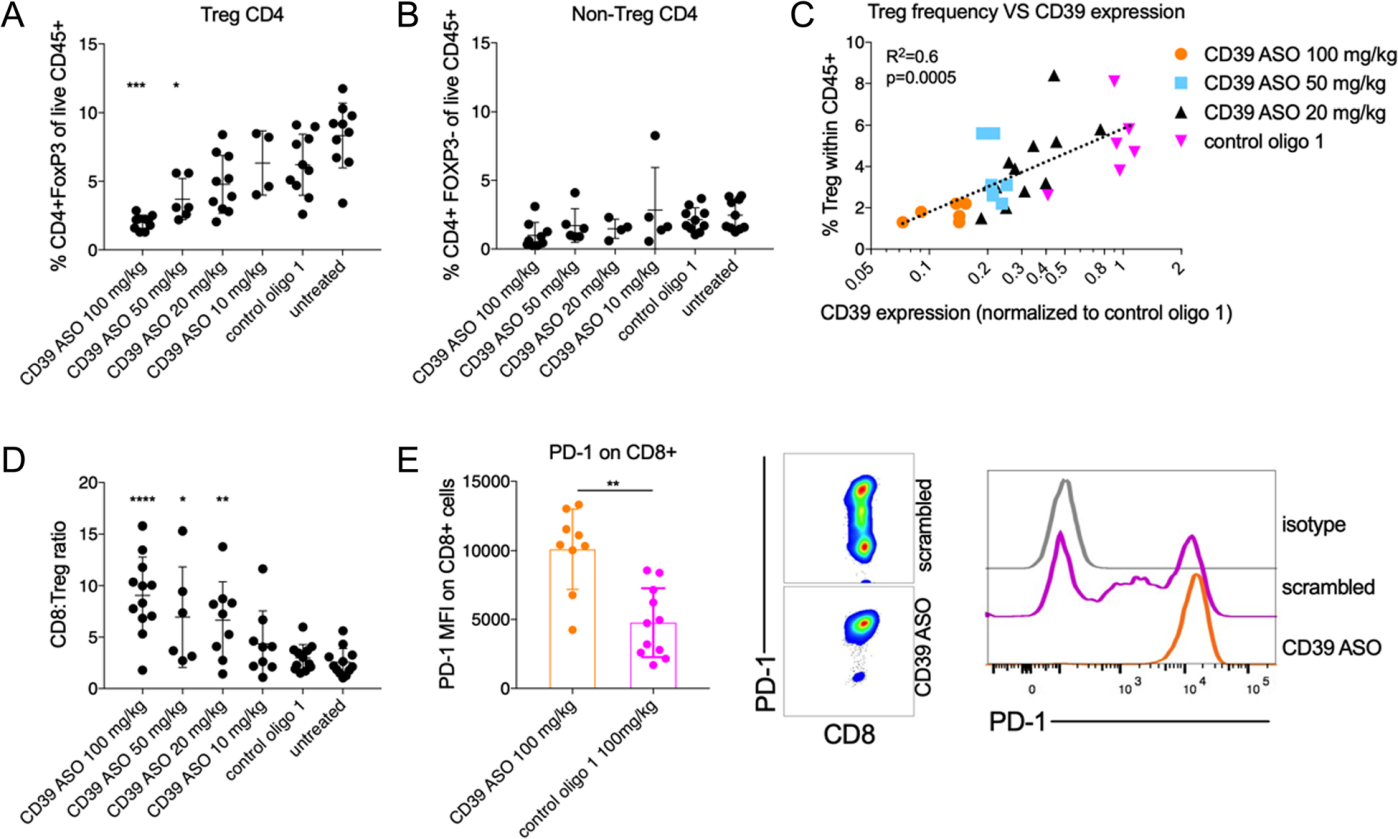
Changes in tumor infiltrating T cell frequencies upon mCD39-specific ASO treatment. Mice bearing homogenous MC38 tumors (between 50 and 80 mm^3^) were injected with CD39 ASO at indicated concentrations, or control oligo 1 (100 mg/kg) on days 1–5. On day 8 or 9 tumors were digested to isolate tumor infiltrating immune cells. Frequency of tumor infiltrating Tregs (live CD4^+^FoxP3^+^CD25^+^) (**a**) and non-Tregs (CD4^+^FoxP3^−^CD25^−^) (**b**) is indicated as percent of live CD45^+^ cells. (**c**) CD39 expression on Tregs is depicted against Treg frequency at the respective CD39 ASO doses. (**d**) Frequency of tumor infiltrating CD8^+^ cells was assessed in tumors of mice treated as per (**a**) and ratio of CD8^+^ cells to Tregs is depicted. (**e**) PD-1 expression (MFI) on tumor infiltrating CD8^+^ cells of CD39 ASO and control oligo 1 -treated mice was assessed by flow cytometry. Representative dot plots (middle) and overlapping histograms (right) depict the increase in PD-1 expression on CD8^+^ cells of CD39 ASO treated tumors compared to control oligo 1. In all cases each data point represents a mouse. Pooled data from two to three independent repeats. Error bars indicate SD. Asterisks indicate significant differences compared to control oligo 1 group

Tumor infiltrating T cell populations were analyzed by flow cytometry after treatment with mCD39-specific or control oligo 1. A dose-dependent reduction in Treg cell numbers (and frequency) was observed in tumors of mice treated with mCD39-specific ASO while non-Treg CD4^+^ T cell numbers remained unchanged (Fig. 5a, b). Particularly, we noticed that the decrease in Treg frequency was positively correlated with the extent of CD39 expression (Fig. 5c). CD8^+^ T cell frequency did not change upon treatment with the ASO (Additional file 6: Figure S4A). As a consequence of altered Treg cell frequency, the CD8^+^ T cell to Treg ratio was substantially higher in the tumors of mCD39 ASO treated mice (Fig. 5d). This indicates a potential skewing from an immunosuppressive to a pro-inflammatory tumor microenvironment. Accordingly, CD8^+^ T cells in the mCD39-specific ASO treated tumors expressed higher levels of PD-1 (Fig. 5e) and CD25 (Additional file 6: Figure S4B), which may likely reflect increased T cell activation. Compared to the control oligo 1 group, the majority of CD8^+^ T cells in mCD39-specific ASO group were PD-1 positive (40% vs. 96%, Fig. 5 e, middle).

### mCD39-specific ASO treatment combination with anti-PD-1 antibody leads to anti-tumor efficacy

**Fig. 6:**
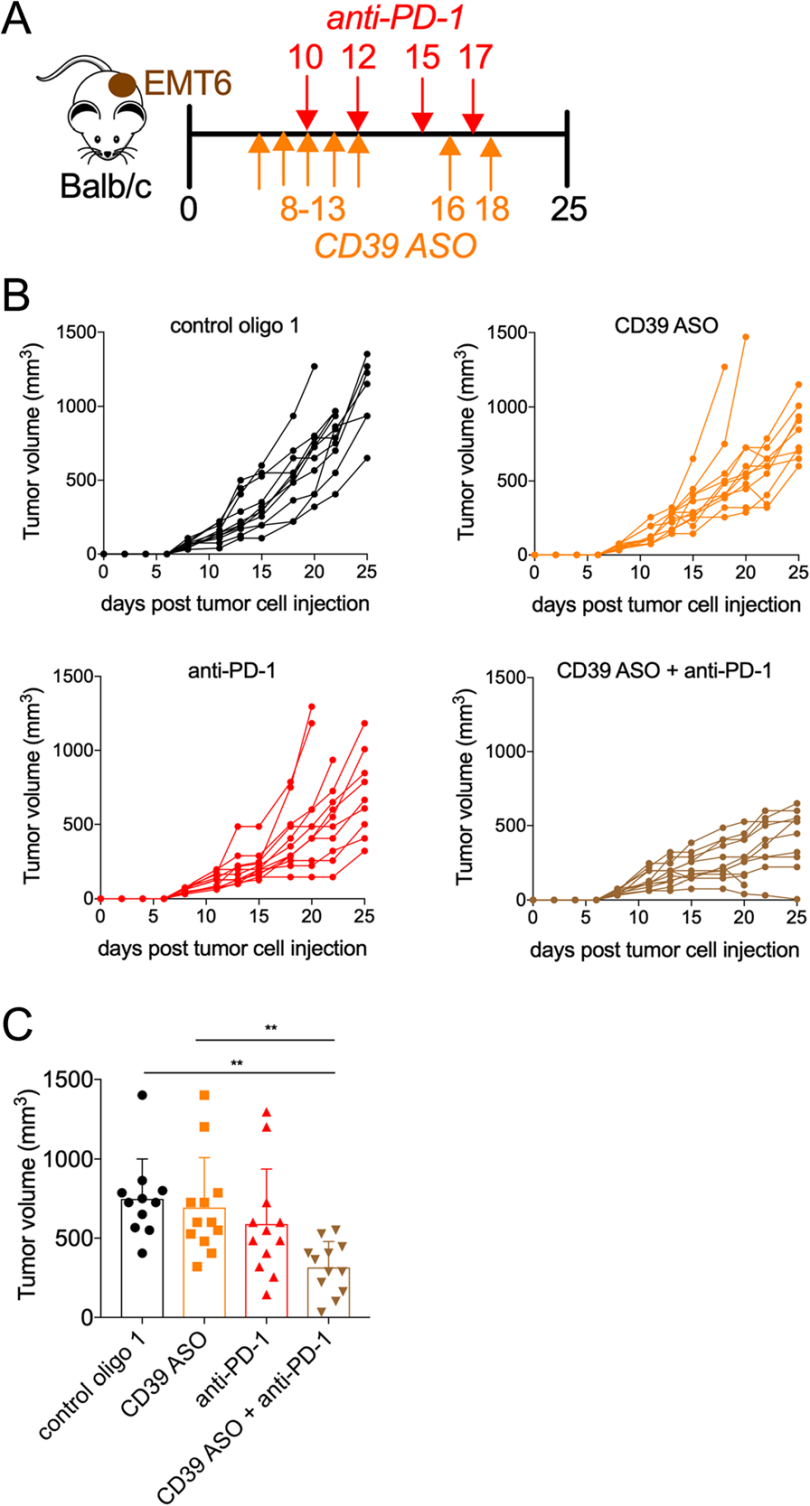
Therapeutic efficacy of CD39 ASO in combination with anti-PD-1 antibody. (**a**) Mice were injected intramammary with 250,000 EMT6 murine breast cancer cells. Once tumors reached 50–80 mm^3^ (Day 8) mice were injected with CD39 ASO, control oligo 1 and/or anti-PD-1 mAb (all i.p.) on indicated days. Mice were treated with CD39 ASO on five days in the first week and on two days in the second week. Tumor growth is depicted as spider plots (**b**) and as bar graph indicating cumulative tumor volume on day 25 post cell injection (**c**). Also included in (**c**) are euthanized mice reaching termination criteria (tumor volume above 1200 mm^3^) before day 25. In all cases each data point represents a mouse. Pooled data from two independent repeats. Error bars indicate SD. Asterisks indicate significant differences compared to control oligo 1 group

Targeting PD-1 on T cells represents a potential strategy to reinvigorate T cell function and offers a rational combination approach. We therefore tested the therapeutic efficacy of systemic CD39 ASO treatment in combination with anti-PD-1 antibody. To this end, we employed an orthotopic breast cancer tumor model by injecting EMT6 cells into the mammary gland (Fig. 6a). While treatment with mCD39-specific ASO resulted in a non-significant reduction in tumor size, combined treatment of mCD39-specific ASO and anti-PD-1 antibody led to significant reduction in tumor burden compared to vehicle control or mCD39-specific ASO monotherapy (Fig. 6b, c); one out of 12 mice rejected the tumor. These data provide a clinically relevant proof-of-principle for the therapeutic utility of CD39 targeting ASO which can be effectively combined with antibodies targeting PD-1.

## Discussion

To further enhance the benefit of cancer immunotherapy, strategies targeting mechanisms of tumor immune evasion and/or resistance are of high clinical relevance. There is increasing evidence that extracellular adenosine generated by the ectonucleotidases CD39 and CD73 is a key metabolite that negatively regulates anti-tumor immunity [13]. In our study, we therapeutically target the expression of CD39 by ASOs. We demonstrate that CD39-specific ASOs reinstate ATP supply through potent and sustained suppression of CD39 expression on T cells, which improves T cell proliferation. Systemic treatment of CD39-specific ASO in vivo resulted in a substantial dose-dependent reduction (> 50%) of CD39 protein mainly in TAMs and Tregs, the latter correlating with an efficient reduction in Treg frequency. Improved tumor infiltrating CD8^+^ T cell to Treg density reflects reinvigorated anti-tumor immunity which could be boosted by anti-PD-1 antibody to induce therapeutic efficacy.

Inhibitors of the CD39/CD73 pathway currently developed for clinical application are mainly composed of monoclonal antibodies and small molecule inhibitors [14]. We report here an entirely novel approach based on LNA Gapmer ASOs. While antibodies and small molecules may modulate the activity of already expressed targets, ASOs prevent the formation of the target protein by degradation of its mRNA. Chemically modified ASOs have enhanced stability and half-life, which may result in longer-lasting effects in vitro and in vivo [15]. Furthermore, ASOs exhibit high target specificity, which reduces side effects and due to their low molecular weight exhibit better tumor penetration.

We and others have demonstrated that tumor infiltrating T cells express high levels of CD39 in human and murine tumors [16, 17], and that the CD39-mediated release of excess adenosine generated from ATP leads to the suppression of T cell function in an autocrine manner. In agreement, we show that addition of extracellular ATP to CD39 expressing human CD8^+^ T cells considerably reduced their proliferation most likely due to the generation of adenosine by CD73, which can also be expressed by T cells [18]. In line with this, we demonstrate that human CD39-specific ASOs potently target CD39 expression on T cells in vitro and that the reduction of T cell proliferation was reverted by ASO-mediated suppression of CD39 expression. We therefore hypothesize that systemic treatment of cancer patients with CD39 ASO firstly could lead to reduced expression of CD39 in distinct tumor infiltrating immune cell populations. Secondly, this reduction in CD39 expression in turn can lead to a decreased degradation of ATP in the tumor microenvironment. As shown here in vitro, this could lead to increased proliferation of CD8^+^ effector T cells, thereby potentially improving anti-tumor T cell responses.

To validate our findings in vivo, we utilized an ASO specifically targeting mouse CD39 in immunocompetent murine tumor models. In agreement with published findings [6, 19–21], CD39 was considerably expressed on a range of different tumor infiltrating immune cells. Of note, we noted the highest expression levels on TAMs, while tumor cells and B cells showed only moderate CD39 expression. Strikingly, systemic treatment of MC38 tumor bearing mice with a mouse-specific CD39 ASO potently suppressed CD39 protein expression on tumor infiltrating CD4^+^ Tregs and TAMs, but not on CD8^+^ T cells. Along the same line, the frequency of T cells in the tumor remained unchanged by CD39 ASO treatment. It is known that different cell types have different sensitivities to gymnotic delivery [11, 22], which may explain the observed differences in CD39 knockdown efficacy in different cell types. Indeed, additional extensive investigations are required to dissect cellular tropism and kinetics of ASOs in vivo. Furthermore, additional stimuli beyond CD39 inhibition may be necessary to provoke measurable infiltration with T cells as shown by others before [23, 24].

Tregs are well known to promote tumor progression by suppressing anti-tumor immunity [25, 26]. Various murine tumor models are known to be infiltrated by Tregs, and their depletion often improves anti-tumor immune responses [27–29]. Furthermore, increased numbers of Tregs correlate with poor prognosis in various types of human cancers [26, 30]*.* Of note, Tregs in tumor-bearing hosts are known to express CD39 [31]. While almost all CD4^+^CD25^+^ cells in mice were reported to express high CD39 levels, only a subset of Foxp3 regulatory effector/memory-like T (T_REM_) cells stain positive for CD39 in humans [31]. Of particular interest, head and neck squamous cell carcinoma (HNSCC) patients were characterized by increased numbers of CD39^+^ Tregs, which hydrolyzed ATP at higher rates, and produced higher levels of adenosine than Tregs from healthy individuals [32]. This is consistent with data gained from tumor models in mice demonstrating that Tregs undergo apoptosis via oxidative stress in the tumor microenvironment and thereby release large amounts of ATP that is converted into adenosine via CD39 and CD73 [33]. In contrast, Tregs from CD39-knock-out (KO) mice failed to generate adenosine and were therefore unable to suppress proliferation of CD4^+^ non-Treg cells in vitro [34]. In our study, ASO-mediated knockdown of CD39 correlated strongly with Treg frequencies in the tumor. While A2AR stimulation promotes the formation of FoxP3^+^ Treg [35, 36], CD39 inhibition may therefore, through reduced adenosine formation and decreased A2AR stimulation, reduce intratumoral Treg frequency after treatment with CD39-specific ASOs. Ultimately, this leads to a higher effector CD8^+^ T cell to Treg ratio, which is associated with improved clinical outcome [37]. We found that PD-1 and CD25 expression on T cells, which likely reflects T cell activation, was increased in mCD39-specific ASO treated animals. Although, assessment of additional markers is necessary to confirm T cell activation upon CD39 ASO treatment. Nevertheless, given the increased expression of PD-1 we speculated that a potential synergy with PD-1 blocking antibodies may be achieved through non-redundant but complementary mechanisms: CD39 ASOs antagonize CD39 expression, reduce Treg frequency and shift the balance towards effector T cells, the latter being further reinvigorated by PD-1 blockade. This strategy is currently pursued in early clinical trials in advanced solid tumors evaluating CD73 or A2a blockade in combination with PD-1/PD-L1 inhibitors (NCT02503774 and NCT02655822).

In conclusion, we demonstrate that ASOs targeting CD39 are able to achieve potent target suppression in relevant cell types in vitro and in vivo and induce potent anti-tumor effects in combination therapy with immune checkpoint inhibitors. Taken together, we developed an innovative immunotherapeutic tool that may potentially improve treatment options for cancer patients in the future.

## Additional files

Additional file 1: **Table S1.** IC_50_-values of selected hCD39 ASOs of first screening round in HDLM-2 cells (DOCX 13 kb)

Additional file 2: **Figure S1.** hCD39-specific ASO inhibit degradation of extracellular ATP in a human Burkitt’s lymphoma cell line. (DOCX 662 kb)

Additional file 3: **Table S2.** Information on mouse-specific CD39 ASOs (DOCX 15 kb)

Additional file 4: **Figure S2.** mCD39 specific ASOs show potent knockdown in the murine B cell lymphoma cell line A20. (DOCX 503 kb)

Additional file 5: **Figure S3.** CD39 expression on T cells and tumor cells after treatment with mCD39-specific ASO. (DOCX 918 kb)

Additional file 6: **Figure S4.** Intratumoral CD8+ T cell frequency and CD25 expression after treatment with mCD39-specific ASO. (DOCX 326 kb)

## Abbreviations

ASO: Antisense oligonucleotide
DC: Dendritic cells
G-MDSC: Granulocytic myeloid derived suppressor cell
LNA: Locked nucleic acids
M-MDSC: Monocytic myeloid derived suppressor cell
NK: Natural killer
TAM: Tumor associated macrophages
TDLN: Tumor draining lymph node
Treg: Regulatory T cells
