# Supplementary material for: Antisense oligonucleotide targeting CD39 improves anti-tumor T cell immunity
Source: J Immunother Cancer. 2019 Mar 12;7:67. doi: 10.1186/s40425-019-0545-9 (PMC6419472; doi:10.1186/s40425-019-0545-9)
Supplement: Supplementary file 1 — Table S1. IC50-values of selected hCD39 ASOs of first screening round in HDLM-2 cells (DOCX 13 kb) [file 40425_2019_545_MOESM1_ESM.docx]

| **ASO** | **IC_50_ [nM]** |
| --- | --- |
| **A04019H** | 40.0 |
| **A04040H** | 25.3 |
| **A04042H** | 60.9 |
| **A04044H** | 46.3 |
| **A04045H** | 66.8 |

**Table S1**: IC_50_-values of selected hCD39 ASOs of first screening round in HDLM-2 cells
