# Supplementary material for: Antisense oligonucleotide targeting CD39 improves anti-tumor T cell immunity
Source: J Immunother Cancer. 2019 Mar 12;7:67. doi: 10.1186/s40425-019-0545-9 (PMC6419472; doi:10.1186/s40425-019-0545-9)
Supplement: Supplementary file 2 — Figure S1. hCD39-specific ASO inhibit degradation of extracellular ATP in a human Burkitt’s lymphoma cell line. (DOCX 662 kb) [file 40425_2019_545_MOESM2_ESM.docx]

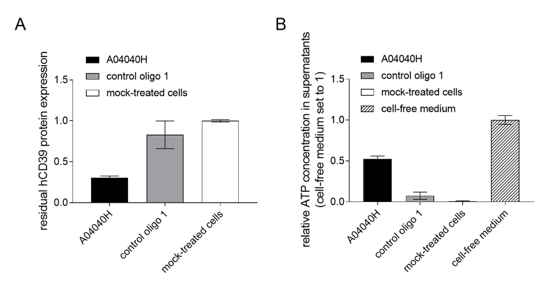


**Figure S1: hCD39-specific ASO inhibit degradation of extracellular ATP in a human Burkitt´s lymphoma cell line.**

JIYOYE cells were treated with the hCD39-specific ASO A04040H or the control oligo 1 at 5 µM for a total treatment time of six days. hCD39 protein expression was analyzed by flow cytometry. **(A)** Residual hCD39 expression relative to mock-treated cells (set as 1) is depicted. For analysis of the capacity to degrade extracellular ATP, 2 µM of ATP was added to cells or cell-free medium. **(B)** Relative ATP concentrations in cell supernatants in relation to cell-free medium (set as 1) were determined after 30 min of incubation with ATP**.** Data is shown of an experiment as mean of six wells +/- SD.
