# Supplementary material for: Antisense oligonucleotide targeting CD39 improves anti-tumor T cell immunity
Source: J Immunother Cancer. 2019 Mar 12;7:67. doi: 10.1186/s40425-019-0545-9 (PMC6419472; doi:10.1186/s40425-019-0545-9)
Supplement: Supplementary file 3 — Table S2. Information on mouse-specific CD39 ASOs (DOCX 15 kb) [file 40425_2019_545_MOESM3_ESM.docx]

| **ASO ID** | **Length** | **Sequence** |
| --- | --- | --- |
| A04001MR | 15 | +A*+G*+T*A*A*T*C*C*A*C*C*C*+A*+T*+A |
| A04002MR | 15 | +G*+A*+T*C*C*A*A*A*G*C*G*C*+C*+A*+A |
| A04003MR | 15 | +G*+T*+T*C*G*T*A*G*T*C*T*C*+C*+A*+G |
| A04004MR | 15 | +C*+T*+G*T*T*C*G*T*A*G*T*C*+T*+C*+C |
| A04005MR | 15 | +G*+G*+T*G*G*C*A*C*T*G*T*T*+C*+G*+T |
| A04006MR | 15 | +G*+T*+T*A*T*A*G*C*C*T*T*G*+C*+A*+G |
| A04007MR | 15 | +C*+A*+C*A*T*T*A*G*C*T*G*C*+A*+C*+G |
| A04008MR | 15 | +C*+C*+T*A*G*T*T*G*T*G*T*A*+T*+A*+C |
| A04009MR | 16 | +G*+T*+A*C*A*G*G*T*T*G*G*T*G*+T*+G*+A |
| A04010MR | 16 | +C*+C*+A*C*T*T*G*T*A*G*A*T*G*+T*+A*+C |
| A04011MR | 16 | +A*+G*+T*A*A*T*C*C*A*C*C*C*A*+T*+A*+G |
| A04012MR | 16 | +G*+C*+C*C*A*G*C*A*G*A*T*A*G*+T*+T*+A |
| A04013MR | 16 | +A*+G*+A*T*C*C*A*A*A*G*C*G*C*+C*+A*+A |
| A04014MR | 16 | +C*+A*+C*T*G*T*T*C*G*T*A*G*T*+C*+T*+C |
| A04015MR | 16 | +T*+G*+G*C*A*C*T*G*T*T*C*G*T*+A*+G*+T |
| A04016MR | 16 | +G*+G*+T*A*C*T*T*C*T*C*C*T*T*+T*+A*+C |
| A04017MR | 16 | +A*+G*+T*T*A*T*A*G*C*C*T*T*G*+C*+A*+G |
| A04018MR | 16 | +C*+G*+T*T*G*C*T*G*T*C*T*T*T*+G*+A*+T |
| A04019MR | 16 | +G*+C*+T*A*T*A*C*T*G*C*C*T*C*+T*+T*+T |
| A04020MR | 16 | +A*+G*+C*A*T*T*T*T*G*G*C*A*T*+C*+A*+C |
| A04021MR | 16 | +C*+C*+T*A*G*T*T*G*T*G*T*A*T*+A*+C*+T |
| A04022MR | 16 | +A*+C*+A*T*T*T*C*T*T*A*C*T*C*+G*+T*+T |
| A04023MR | 17 | +G*+A*+C*C*T*T*T*C*A*C*T*T*G*G*C*A*+T |
| A04024MR | 17 | +C*+C*+C*A*G*C*A*G*A*T*A*G*T*T*+A*+A*+T |
| A04025MR | 17 | +G*+C*+C*C*A*G*C*A*G*A*T*A*G*T*+T*+A*+A |
| A04026MR | 17 | +A*+T*+C*C*A*A*A*G*C*G*C*C*A*A*+A*+G*+G |
| A04027MR | 17 | +T*+C*+G*T*A*G*T*C*T*C*C*A*G*T*+G*+C*+C |
| A04028MR | 17 | +T*+T*+C*G*T*A*G*T*C*T*C*C*A*G*+T*+G*+C |
| A04029MR | 17 | +T*+G*+T*T*C*G*T*A*G*T*C*T*C*C*+A*+G*+T |
| A04030MR | 17 | +G*+G*+T*G*G*C*A*C*T*G*T*T*C*G*+T*+A*+G |
| A04031MR | 17 | +C*+G*+T*T*G*C*T*G*T*C*T*T*T*G*+A*+T*+C |
| A04032MR | 17 | +G*+C*+T*A*T*A*C*T*G*C*C*T*C*T*+T*+T*+C |
| A04033MR | 17 | +T*+A*+C*A*T*T*T*C*T*T*A*C*T*C*+G*+T*+T |

Table S2: Information on mouse-specific CD39 ASOs

+ indicates LNA-modified nucleotides and * indicates PTO linkages
