# Supplementary material for: Antisense oligonucleotide targeting CD39 improves anti-tumor T cell immunity
Source: J Immunother Cancer. 2019 Mar 12;7:67. doi: 10.1186/s40425-019-0545-9 (PMC6419472; doi:10.1186/s40425-019-0545-9)
Supplement: Supplementary file 4 — Figure S2. mCD39 specific ASOs show potent knockdown in the murine B cell lymphoma cell line A20. (DOCX 503 kb) [file 40425_2019_545_MOESM4_ESM.docx]

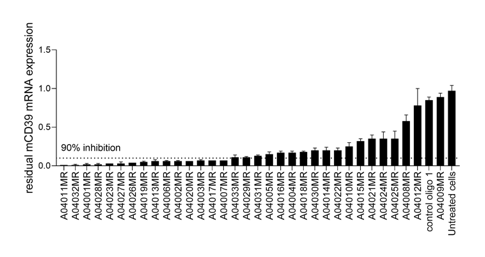


**Figure S2**: **mCD39 specific ASOs show potent knockdown in the murine B cell lymphoma cell line A20.**

A20 cells were treated for three days with mCD39 specific ASOs at a final concentration of 10 µM. The mRNA expression of HPRT1 and CD39 were analyzed and CD39 values were normalized to HPRT1 values. Residual CD39 expression relative to untreated cells (set to 1) is depicted. The mean of triplicate wells +/- SD is shown.
