# Supplementary material for: Antisense oligonucleotide targeting CD39 improves anti-tumor T cell immunity
Source: J Immunother Cancer. 2019 Mar 12;7:67. doi: 10.1186/s40425-019-0545-9 (PMC6419472; doi:10.1186/s40425-019-0545-9)
Supplement: Supplementary file 5 — Figure S3. CD39 expression on T cells and tumor cells after treatment with mCD39-specific ASO. (DOCX 918 kb) [file 40425_2019_545_MOESM5_ESM.docx]

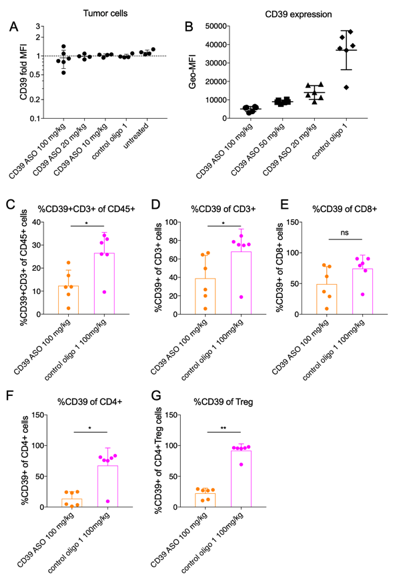


**Figure S3: CD39 expression on T cells and tumor cells after treatment with mCD39-specific ASO.**

Mice bearing palpable tumors (50-80 mm3) were injected i.p with the indicated doses of CD39 ASO or with 100 mg/kg of control oligo 1. Day 9 post ASO injection tumors were digested and CD39 expression on CD45^-^ tumor cells **(A)** or tumor infiltrating CD4^+^FOXP3^+^CD25^+^ Tregs (**B)** or was assessed by flow cytometry. Data in A is geometric mean fluorescence intensity of CD39 whereas data in B is represented as fold-change in CD39 MFI compared to control oligo. (**C-G**) Frequency of CD39^+^CD3^+^ cells amongst live CD45^+^ (C), as well as frequency of CD39^+^ cells amongst CD3^+^ cells (D), CD8^+^ cells (E), CD4^+^ cells (F) and CD4^+^FOXP3^+^CD25^+^ Tregs (G) was also assessed from the same MC38 tumor digests as A,B isolated from mice treated with either CD39 ASO 100 mg/kg or control oligo 1. In all cases each data point represents a mouse. Pooled data from two independent repeats. Error bars indicate SD.
