# Supplementary material for: Antisense oligonucleotide targeting CD39 improves anti-tumor T cell immunity
Source: J Immunother Cancer. 2019 Mar 12;7:67. doi: 10.1186/s40425-019-0545-9 (PMC6419472; doi:10.1186/s40425-019-0545-9)
Supplement: Supplementary file 6 — Figure S4. Intratumoral CD8+ T cell frequency and CD25 expression after treatment with mCD39-specific ASO. (DOCX 326 kb) [file 40425_2019_545_MOESM6_ESM.docx]

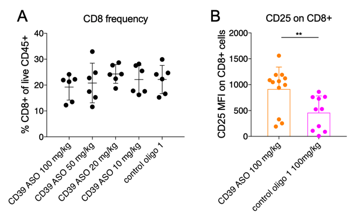


**Figure S4**: **Intratumoral CD8+ T cell frequency and CD25 exoression after treatment with mCD39-specific ASO.**

Mice bearing palpable tumors (50-80 mm3) were injected i.p with the indicated doses of CD39 ASO or with 100 mg/kg of control oligo 1. (**A**) CD8^+^ T cell frequency, expressed as percent of live CD45+ cells, infiltrating into the tumor day 9 post CD39 ASO treatment is depicted. (**B**) CD25 expression was assessed on CD8^+^ T cells and expressed as MFI. Each data point represents a mouse. Pooled data from two independent repeats. Error bars indicate SD.
